# A novel case of 16q22.3 duplication syndrome in a child with overgrowth: case report and literature review

**DOI:** 10.1186/s12920-023-01716-3

**Published:** 2023-12-04

**Authors:** Antonino Moschella, Anna Paola Capra, Domenico Corica, Giorgia Pepe, Silvia Di Tommaso, Ester Sallicandro, Malgorzata G. Wasniewska, Silvana Briuglia, Tommaso Aversa

**Affiliations:** 1 Department of Biomedical, Dental, Morphological and Functional Imaging Sciences, “BIOMORF”, Unit of Genetics and Pharmacogenetics, University of Messina, Messina, Italy; 2 Department of Chemical, Biological, Pharmaceutical and Environmental Sciences, University of Messina, Messina, Italy; 3 Department of Human Pathology of Adulthood and Childhood “G. Barresi”, Unit of Paediatrics, University of Messina, Messina, Italy; 4 Laboratory of Medical Genetics, Translational Cytogenomics Research Unit, “Bambino Gesù” Children Hospital, IRCCS, Rome, Italy

**Keywords:** 16q22, Distal duplication of chromosome 16q, CNV, Intellectual disability, Overgrowth, Obesity, Case report

## Abstract

**Background:**

Distal chromosome 16 duplication syndrome (also known as 16q partial trisomy) is a very rare genetic disorder recently described in few clinical reports. 16q trisomy is generally associated with a multisystemic phenotype including intrauterine growth restriction (IUGR), brain and cardiac defects, intellectual disability (ID) and an increased risk of both prenatal and postnatal lethality. Smaller copy number variants (CNV) within the 16q region create partial trisomies, which occur less frequently than full trisomy 16q.

**Case presentation:**

We present the clinical case of a 12-years-old male with a 16q22.3q24.1 de novo heterozygous duplication whose phenotype was characterized by ID, facial dysmorphisms, stature and weight overgrowth. To date, only five other cases of this syndrome have been reported in scientific literature, and none of them comprised overgrowth.

**Conclusions:**

Our case report highlights the great heterogeneity in clinical manifestations and provides new evidence for better defining the phenotypic picture for smaller 16q distal CNVs, suggesting unusual features.

**Supplementary Information:**

The online version contains supplementary material available at 10.1186/s12920-023-01716-3.

## Background

Distal chromosome 16 duplication syndrome, also known as 16q partial trisomy, is a very rare genetic disorder resulting from the partial trisomy of the long arm of chromosome 16. 16q trisomy is generally associated with a multisystemic phenotype including intrauterine growth restriction (IUGR), stunted growth in height and weight, brain and cardiac defects, intellectual disability (ID) and an increased risk of both prenatal and postnatal lethality.

Smaller chromosomal copy number variants (CNVs) within the 16q region create partial trisomies, which occur less frequently than full trisomy 16q. However, this syndrome is not well-characterized in the literature due to the limited clinical and prognostic information available [1].

Previous works suggested an important role of this chromosomal region in neurological development, and partially overlapping duplications are associated with a similar phenotype in few other cases reported in the scientific literature. These patients showed a core phenotype of developmental delay (DD) / ID and midface hypoplasia associated with incomplete penetrance and high variable expression in both inter and intrafamilial cases [1].

Facial dysmorphisms include high and prominent forehead, epicanthic folds, dysplastic ears, broad/depressed nasal bridge, malar hypoplasia, narrow and arched palate, thin upper lip vermilion, micrognathia, and hand/feet anomalies. Also, cardiac defects, genitourinary malformations, and vertebral anomalies can be associated. Thrombocytopenia and recurrent infections have also been reported.

To date, in addition to the few patients characterized by chromosomal microarray analysis (CMA) reported in the literature [1], other 13 cases encompassing the shared region have been reported in the DECIPHER repository [2].

Here we report the clinical case of a young male with distal microduplication included in the 16q22.3q24.1 region with a peculiar phenotypic picture characterized by stature and weight overgrowth as unusual features.

## Case presentation

**Fig. 1:**
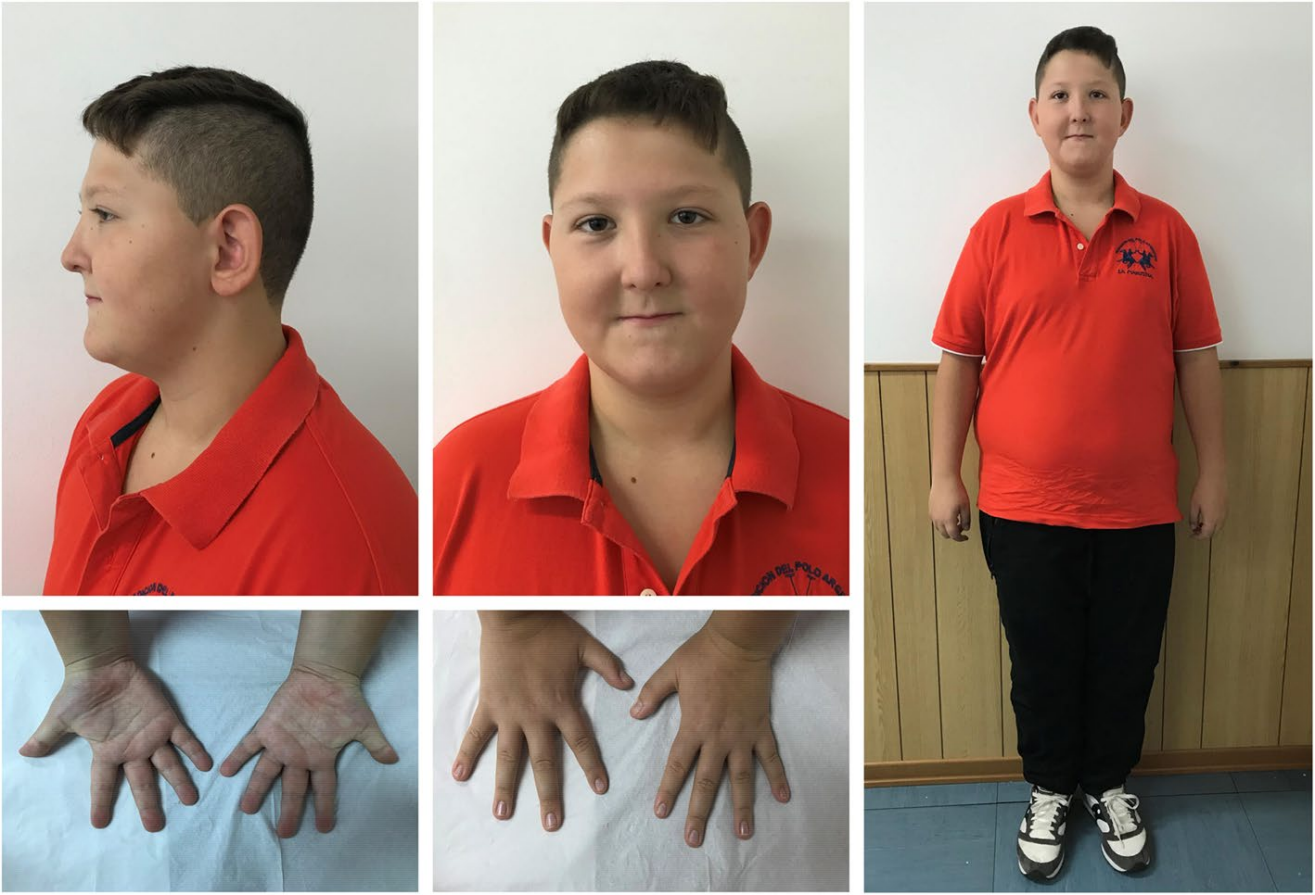
Photographs of our patient showing dysmorphic facial features, in particular: sparse eyebrows, epicanthus, hypertelorism, midface hypoplasia and thin lips. Absence of 5th finger clinodactyly in both hands

Our patient was born at 36 weeks of gestation by a twin pregnancy of a 28-year-old Italian woman. Weight at birth was 2450 g. He presented a clinical history of complications during the pregnancy for maternal pre-eclampsia and chirurgical partum, no hypotonia or feeding difficulties at birth. The family history was totally negative for ID or neurodevelopmental disorders, the twin brother was healthy. Growth charts showed height growth above the mean for age and target stature and weight growth significantly above the mean for age from the early years of life and even in the neonatal period. At the age of 3 years, speech delay and psychomotor delay were diagnosed. Simultaneously, because of minor dysmorphic features, some genetic investigations have been performed, such as karyotype and molecular analysis for Kabuki Syndrome, both resulted normal. The twin brother also underwent karyotype analysis which resulted normal. At the age of 6 years, cleft palate was surgically corrected. During childhood, several specialist assessments were performed and the patient was followed up due to severe early-onset obesity. Audiological tests, echography of the abdomen and heart, and brain MRI were performed, excluding the presence of any major abnormality or functional disorder. At the time of our first outpatient visit, at the age of 12 years, the patient’s phenotype was characterized by severe obesity (BMI + 3.2 SD), high stature (height + 1.39 SD), and minor dysmorphic facial traits including sparse eyebrows, epicanthus, hypertelorism, midface hypoplasia, and thin lips. Occipital-frontal head circumference (OFC) was 55 cm (+ 0.54 SD). Bone age was found to be about 13 years, but testicular volume was still prepubertal in size according to Tanner stage. Our patient was cooperative and calm on physical examination (Fig. 1). He underwent several clinical and laboratory investigations to rule out secondary causes of obesity, including endocrinopathies. To date, no hormonal disorder has been identified. Haematochemical investigations documented a picture of fasting hyperinsulinaemia (insulin 33 IUI/mL, but not after oral glucose load), with normal fasting glycaemia (86 mg/dL), normal glucose tolerance (glycaemia 110 mg/dL after 120' after oral glucose load) and glycated haemoglobin (5.5%). Levels within normal limits were documented for TSH (3.860 IU/mL), FT4 (16.40 pmol/L), FT3 (4.69 pg/mL), IGF-1 (198 ng/mL), cortisolemia (14.48 ug/dL), ACTH (67. 3 pg/mL), 24-h cortisoluria (120 mcg/24 h), vitamin D (28 ng/mL), calcemia (10 mg/dL), phosphoremia (4.1 mg/dL), PTH (20 pg/mL), alkaline phosphatase (200 U/L). Screening for coeliac disease was negative. Gonadotropins were compatible with the early pubertal stage reached (FSH 7.54 mUI/mL; LH 2.02 mUI/mL), as were testosterone levels (15.5 ng/dL). The total cholesterol (190 mg/dL) and LDL-cholesterol (100 mg/dL) were at the high limit of the normal range for age; HDL-cholesterol (57 mg/dL) and triglycerides (55 mg/dL) were within the normal range. 24 h arterial pressure monitoring was normal. At the last endocrinological assessments, at the age of 14, the boy appeared to have a spontaneous onset of puberty (testicular volume from 3 to 5 cc bilaterally; bone age 14.6 years). Severe obesity was persistently present (BMI + 3.2 SDS), whereas a slowing of height growth rate (2.6 cm/6 months) was recorded with stature (+ 0.6 SDS) still above the target height (+ 0.3 SDS).

At present, the patient is also being followed up for mild to moderate ID with speech impairment and is undergoing neuropsychiatric training and speech therapy with good results.

## Genetic analysis

**Fig. 2:**
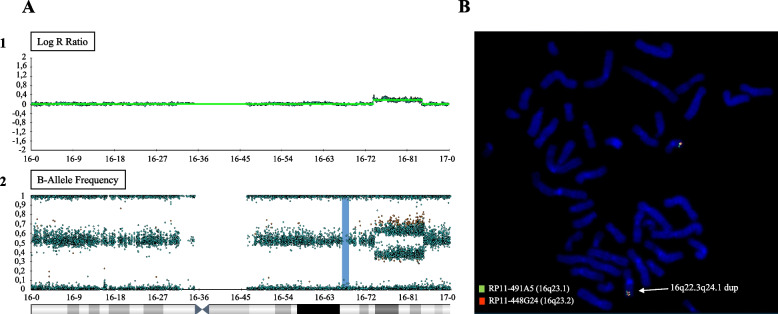
Cytogenetic analyses results. **A**: Illumina SNParray showing copy gain in 16q22.3q24.1 by mean of Log R ratio (1) and B-Allele frequency (2). **B**: image of FISH on metaphases obtained by lymphocyte cultures with the arrow indicating the duplicated chromosomal region

Our patient underwent molecular cytogenetics analyses as routine diagnostic procedures. Peripheral blood samples of the patient and his parents were collected and the informed consent was signed by patient's parents. According to standard procedures, Chromosomal Microarray Analysis (CMA) was performed on DNA extracted from peripheral blood using Infinium CytoSNP-850 K BeadChip (Illumina, San Diego, California) at an average resolution of 100 kb. Array scanning data were generated by the NextSeq 550 system (Illumina) and results were analyzed by Bluefuse Multi software. Nucleotide designations were assigned according to the hg19/GRCh37 assembly of the human genome. Validation of the genomic rearrangement was performed by FISH (fluorescence in situ hybridization) on metaphases obtained by lymphocyte cultures. RP11-491A5 (16q23.1) and RP11-448G24 (16q23.2) BAC (Bacterial artificial chromosome) clones were used for this purpose. CMA analyses and FISH testing revealed a de novo heterozygous duplication of 16q22.3q24.1 chromosomic region, about 10.5 Mb in size, spanning from position 74,005,024 to 84,546,108 (GRCh37), classifiable as a pathogenic CNV according to American College of Medical Genetics and Genomics (ACMG) guidelines. The duplication resulted to be inserted in tandem as revealed by the alternating of the signals (green-orange::green-orange) (Fig. 2). The CMA also showed a heterozygous deletion in 5q14.3 (90,540,522–90653597), 113 kb in size, paternally inherited and classifiable as a CNV of uncertain significance (VoUS).

## Discussion and conclusions

Trisomy of 16q is usually associated with a multisystemic phenotype including IUGR, stunted growth in height and weight, brain and cardiac defects, and an increased risk of both prenatal and postnatal lethality. Smaller CNVs within the 16q region determine partial trisomies, which occur less frequently than full trisomy 16q. Currently, 5 cases of distal 16q duplication have been reported in the scientific literature [1]. Previous works included 3 males and 2 females. The authors described the core phenotype for distal duplications of 16q, highlighting ID and midface hypoplasia as constant features (Table 1). Furthermore, they suggested highly variable expressivity, even in intrafamilial cases, and hypothesized sex differences for a potential association with psychiatric disorders predisposition in female patients [1, 3–5].

**Table 1:** 16q22.3 duplication syndrome cases

	**Patient 1 **[6]	**Patient 2 **[4]	**Patient 3 **[5]	**Patient 4 **[1]	**Patient 5 **[1]	**Patient 6 (this report)**
D**uplication Size**	6.1 Mb	8.3 Mb	17.6 Mb	8.85 Mb	8.85 Mb	10.5 Mb
**Cytoband**	16q22.1q23.1	16q22.1q23.1	16q22.3q24.3	16q22.3q23.3	16q22.3q23.3	16q22.3q24.1
**Sex**	M	M	F	M	F	M
**Height**	‐3 SD	‐2 SD	Not reported	0 SD	+ 1 SD	+ 1.39 SD
**Weight**	‐2 SD	‐2.5 SD	Not reported	‐1 SD	‐1 SD	> + 3 SD
**OFC**	‐0.6 SD	< − 2 SD	Not reported	‐2.6 SD	+ 2 SD	+ 0.54 SD
**ID**	+	+	‐	+	‐	+
**Seizures**	+	‐	‐	+	‐	‐
**Dysmorphic features (including midface hypoplasia)**	+	+	‐	+	‐	+
**Congenital anomalies**	+	+	‐	+	‐	+
**Neurologic Features**	+	+	+	+	‐	-
**Psychiatric Features**	‐	‐	+	‐	+	-
**Other**	5th finger clinodactyly, flat foot, wide gait, cryptorchidism, mild anemia, vesicoureteric reflux	Vision loss, hypothyroidism	None	5th finger clinodactyly, left toes with outward deviation	5th finger clinodactyly	Cleft palate, obesity, pubertal delay

Comparison of the clinical features of the six known patients carrying duplications of the16q22.3 region, including the patient presented in this paper. Adapted from Gunther et al. [1]

Here we report a novel case of a duplication involving the 16q22.3q24.1 region, identified in a male patient sharing significant clinical characteristics with the previously described cases.

**Fig. 3:**
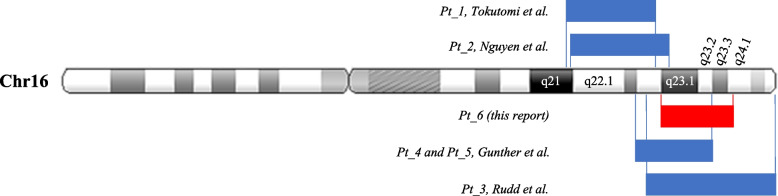
Chromosome schematic of the duplication in our patient (red box) referenced with the mentioned in the literature (blue boxes) to compare size and location

We explored the chromosomal overlapping region involved in the 5 known cases for comparison with our patient's CMA result to point out a suggestive critical region for 16q distal duplication syndrome. The involved regions spanning from 6.1 Mb to 17.6 Mb are differentially positioned in relation to the 16q22.3q24.1 region (Fig. 3). Three of these patients (two of them were blood relatives) presented copy number gains overlapping quite at the same start point, confirming a potential causality for the observed clinical features, although, in these 3 subjects, we cannot definitively conclude for a peculiar phenotype common with that of our patient. The other two smaller duplications (6.1 Mb to 8.3 Mb) map at 16q22.1q23.1, a slightly more proximal region, and both patients differ from the other three and from our patient. For example, patients 1 and 2 present a growth retardation, which is not evident in the other cases and in our patient, as he presents an overgrowth (Table 1). Therefore, in view of the shared phenotypic features, we might assume that the critical region of this distal 16q duplication syndrome mainly involves regions downstream of the q22.3 band. The phenotype of the first two patients could therefore also be ascribed to other genes in more proximal regions. It is interesting to investigate the contribution of genes outside the region to the clinical phenotype, because in this case, despite the limited number of patients, ID, dysmorphic features, congenital anomalies, and neurological manifestations were observed, without psychiatric features.

The 16q22.3q24.1 region spans 10.5 Mb and includes 101 genes. In order to predict the effect of this large copy number gain, we noticed that 17 of 101 genes are reported as morbid genes associated with disease phenotypes, and 13 coding genes resulted in more interesting collected evidence supporting dosage sensitivity. However, triple-sensitivity has not been evaluated yet. All these genes are listed in Additional File 1, including multiple annotations about the duplicated region: chromosome, genes, and integrated clinical information. These data have been generated and adapted from the CNV-ClinViewer database [6].

Overlapping duplications involving the intermediate-distal chromosomal region were compared, documenting that almost all reported rearrangements are significantly larger and associated with more complex phenotypes. Among these cases it is worth mentioning a 47-month-old Brazilian girl with a de novo 22.3 Mb 16q21q24.1 duplication, arr[GRCh37] 16q21q24.1(62586414_84885185) × 3. It is important to note that there are some similar clinical findings to previously discussed cases: facial dysmorphisms, hypotonia with motor milestone delay, cognitive impairment with language delay, epilepsy without brain malformation, 5th finger clinodactyly, vesicoureteral reflux, in association with hearing loss [7]. However, this duplication is significantly larger and more proximally extended than the CNVs described in the other discussed cases, which involved principally the distal 16q region.

In order to better define the variable clinical expressivity of 16q22.3q24.1 duplications, 13 additional individuals from the DECIPHER repository [2] were included in the genomic and clinical comparison (5 females, 7 males, 1 unknown). Patients reported on DECIPHER presented microduplications with partial overlap but with a larger extension and different endpoints. These cases were included in our analysis in order to investigate a possible critical region and its association with the phenotype.

The main clinical features reported in DECIPHER (Patients: 394,947; 394,920; 393,340; 393,290; 367,452; 362,131; 356,957; 349,797; 340,313; 296,404; 285,657; 256,542; 253,240) were hypotonia (5 subjects), ID (4 subjects), hypotelorism (4 subjects), low-set ears (3 subjects), epicanthus (3 subjects), microcephaly (2 subjects), frontal bossing (2 subjects) hearing impairment (2 subjects), atrial septal defect (2 subjects).

In 6 cases the copy number gain resulted segregated as an imbalance arising from a balanced parental rearrangement, in 4 cases was de novo, and in the others unknown. In 10 subjects the 16q duplication was present in association with at least another CNV, involving other chromosomal regions. Clinical features and CMA results of DECIPHER patients are reported in Additional File 2.

Recurrent association with other chromosomal CNVs (68%) could explain additional clinical manifestations such as heart defects, hearing loss, hearing impairment, and facial dysmorphisms (epicanthus, low set ears, frontal bossing, cleft palate), suggesting more complex genotype–phenotype correlations.

We performed a comparison among known cases in the literature, those reported into DECIPHER database and our patient, to identify possible genotype–phenotype correlations in order to further delineate the phenotype of 16q duplication syndrome. All collected patients (*n* = 19) had DD/ID (89%), hypotonia (26%), and hypertelorism (26%), which were confirmed as the most common clinical signs, with no evident sex difference in phenotypic expression.

Moreover, two other cases of segmental trisomy 16q were described in a recent review work, involving respectively the chromosomal regions 16q12.1q23.3 and 16q12.2q24.3. These chromosomal abnormalities were significantly larger and showed a limited overlap in comparison with the region described in our case. Both the patients presented a complex phenotype with congenital anomalies and cognitive impairment resulting in a severe and life-threatening clinical picture, probably due to the extension of the duplication and the involvement of numerous genes [8].

Although our patient shares significant clinical features with the previously reported cases, the peculiarity of this case is the finding of overgrowth, pubertal delay and cleft palate.

A cleft palate was only reported among the clinical features of a Sri Lankan female infant with partial trisomy 16q21➔qter, a result of unbalanced segregation of a maternal balanced translocation t(15;16), in association with craniofacial dysmorphic features and the presence of cardiac and anorectal malformations. [9]. A previous work described a girl with a partial trisomy 16q suffering from obesity and ID. However, this duplication involved the proximal region of the long arm of chromosome 16, encompassing the *FTO* gene and suggesting a possible association with obesity [10].

On the contrary, none of the described cases of distal duplication included obesity. Duplications are not commonly detected in obese patients [11, 12], but the chromosomal region 16q22.1-q24.1 was reported with suggestive linkage association with childhood obesity [13]. Among non-imprinted regions, 16q22 was also reported in suggestive linkage with BMI, supporting the hypothesis that it can be involved in weight regulation and development of obesity [14]. Of note, the short arm of 16 chromosome is known for including a critical region on 16p11.2 for a mirror syndromic phenotype which can show growth restriction in case of duplication and obesity in case of deletion [15]. To date, not enough information are available for defining similar functional consequences of chromosomal aberration at 16q22.3.

At present, it is difficult to identify a single gene within this chromosomal region that may be more likely related to the development of obesity, as well as with psychiatric disorders, mainly because of the limited number of patients described. However, neurocognitive evaluation of our patient did not reveal any relevant psychiatric symptom. Furthermore, though previous reports suggested a novel site of chromosome instability predisposing to rearrangements, our patient’s duplication was de novo.

With regard to the 5q14.3 paternal deletion, this chromosomal aberration is inner to the *LUCAT1* gene. To date, no information is known about haploinsufficiency of this gene and the father was apparently asymptomatic. Larger deletions encompassing the 5q14.3 chromosomal region are associated with a syndromic phenotype with ID, seizures, and brain anomalies [16]. However, the reported patients showed a clinical phenotype which resulted to be essentially related to the haploinsufficiency of *MEF2C*. Some patients showed also vascular malformations in case of other gene involvement (*RASA1*) [17]. None of these genes are located in the chromosomal region described in our case. Thus, according to ACMG recommendations, the microdeletion that has been identified in our patient and his father should be classifiable as a CNV of uncertain significance, suggesting a benign role within the patient phenotype.

In conclusion, in this paper we describe a novel case of 16q distal duplication syndrome. Our report confirms the clinical heterogeneity and provides new evidence for better defining the phenotypic picture for smaller 16q chromosomal rearrangements, associated with ID, even mild, and typical dysmorphic features. Moreover, the clinical picture of our patient was characterized by stature and weight overgrowth, suggesting the possibility of other features within the 16q distal duplication spectrum. To date, only five other cases of this syndrome have been characterized in scientific literature.

Our report confirms the core phenotype described before and can be helpful for further expanding the clinical spectrum of 16q distal duplication syndrome.

### Supplementary Information

**Additional file 1. **

**Additional file 2.**

## Data Availability

All data generated or analyzed during this study are included in this published article and its supplementary information files. Public data included in this study are available in the DECIPHER repository (https://www.deciphergenomics.org), accessed on 1 May 2023. The Additional Tables include multiple annotations and integrated clinical information available publicly and adapted from CNV-ClinViewer (https://cnv-clinviewer.broadinstitute.org), accessed on 20 July 2023.

## Abbreviations

IUGR: Intrauterine growth restriction
ID: Intellectual disability
CNV: Copy number variant
DD: Developmental delay
CMA: Chromosomal microarray analysis
OFC: Occipital-frontal head circumference
BMI: Body mass index
FISH: Fluorescence in situ hybridization
BAC: Bacterial artificial chromosome
ACMG: American College of Medical Genetics and Genomics
VoUS: Variant of uncertain significance
